# Patients’ Experiences With Pregnancy of Unknown Location: A Qualitative Study

**DOI:** 10.1177/23743735261458035

**Published:** 2026-06-10

**Authors:** Rafa Ifthikhar, Kellie E. Schueler, Sheila K. Mody

**Affiliations:** 1 Department of Obstetrics, Gynecology and Reproductive Sciences, 8784 University of California San Diego, La Jolla, CA

**Keywords:** pregnancy of unknown location, early pregnancy, patient experience, qualitative research, patient-centered care

## Abstract

Pregnancy of unknown location (PUL) is a common early pregnancy diagnosis, yet patient experiences are not well understood. This study explored emotions and experiences of individuals diagnosed with PUL. Participants aged 18-45 completed semi-structured interviews, and data were analyzed using thematic analysis. Amongst 30 participants, a diverse range of pregnancy types and intentions were represented. We grouped the data into four findings 1) Participants experienced anxiety and negative emotions related to diagnostic uncertainty 2) Effective coping strategies included social support, resilience, and acceptance. 3) Positive experiences were linked to empathetic, responsive providers. 4) Limited access to outpatient care led many to seek emergency care, often characterized by long waits and lack of privacy. In conclusion, patients with PUL experienced considerable anxiety and negative emotions which persisted across different pregnancy intentions and outcomes. Many considered this to be a major life event. Experiences were improved by personal coping skills and having caring providers. Due to lack of urgent outpatient appointments, many patients sought care in the emergency department which was sometimes associated with worse experiences overall.

## 1. Introduction

Pregnancy of unknown location (PUL) is a common diagnosis, however patients’ experiences with the diagnosis are not fully understood. PUL is a transient diagnosis made when a patient has a positive pregnancy test however no definite intrauterine pregnancy is visualized on ultrasound imaging. A diagnosis of PUL prompts additional follow-up testing including repeated blood draws to trend beta-human chorionic gonadotropin (beta-HCG) and ultrasounds until a final diagnosis is made. Final outcomes of PUL include early intrauterine pregnancy, early pregnancy loss (miscarriage), or ectopic pregnancy.^1^ Studies report that the rate of PUL in patients attending early pregnancy ultrasounds varies between 5-42%.^2^

Patient experiences with diagnoses more downstream of PUL, such as early pregnancy loss and ectopic pregnancy, have been described in the literature.^3,4,5^ However, there are few published quantitative or qualitative studies exploring patients’ experiences with PUL.

Much of the existing knowledge regarding patients’ experiences of PUL is derived from studies conducted in early pregnancy assessment units (EPAUs) in the United Kingdom. These centers provide care specifically for patients experiencing complications in early pregnancy, including PUL. Studies on EPAUs thus far have not specifically focused on PUL and rather include all patients experiencing early pregnancy complications. Prior studies demonstrate patients seeking care at EPAUs report high self-reported anxiety. While anxiety levels decreased over time for patients with certain diagnoses, patients with certain diagnosis had significantly higher anxiety levels 48-72 hours their ultrasound.^6^ Patients seeking care in EPAUs generally had a positive experience; however, negative experiences centered around access to services, sensitive patient management, and patient-provider communication.^7^

Additionally, other diagnoses that require frequent and potentially prolonged follow-up are also associated with negative emotions. For example, a study by Di Mattei et al. sampled patients with gestational trophoblastic disease and found that participants reported significant levels of anxiety and psychological distress. Additionally, the study found that fertility perception is negatively affected by a diagnosis of gestational trophoblastic disease.^8^

Lastly Wu et al. conducted a smaller qualitative study on patients with desired pregnancies diagnosed with PUL. Their study yielded four patient priorities and logistical preferences related to PUL management.^9^

Having a better understanding of patients’ experiences of pregnancy of unknown location can help clinicians adopt a more patient-centered approach to care. Additionally, understandings patients’ perspectives will help inform interventions to improve future patients’ experiences with this diagnosis. The aim of this study was to explore patients’ emotions and experiences with the diagnosis of and follow-up care for PUL. Compared to the Wu et al. study, we aimed to explore patients’ experiences and emotions with the PUL diagnosis more broadly, rather than focusing on patient preferences of PUL management. Our study sample includes a different set of participants as we recruited from family planning clinics to include patients with undesired and ambivalent pregnancy intentions. Qualitative methods were selected as they are well suited to exploring understudied phenomena and understanding patient experiences in areas where limited prior evidence exists. Additionally qualitative methods allowed us to explore the richness and nuance of patients’ experiences that is difficult to observe with quantitative methods.

## 2. Materials and Methods

Participants ages 18-50 years old were recruited between Fall 2022 to Spring 2024 from academic obstetrics and gynecology clinics, an emergency department, and a Planned Parenthood clinic in urban and suburban areas in Southern California. Eligible participants were recruited after being diagnosed with pregnancy of unknown location and were able to consent in English. Participants were recruited until thematic saturation was reached, meaning no new information or themes were observed with additional data collection.^10^ Participants provided their verbal consent prior to each interview. Each participant completed a semi-structured virtual interview through videoconferencing. The interview guide included open-ended questions on patients’ experience with the PUL diagnosis including emotions, feelings, coping and support, impact, knowledge, and negative and positive aspects of the experience (Appendix 1). The interview guide remained the same throughout the data collection process, and questions were phrased identically to all participants. To better characterize our sample each participant was sent a link to a basic demographics survey on REDCap after completing the interview.^11,12^ Participants were compensated with a $20 electronic gift card.

Interviews were audio recorded and transcribed. All identifying information was removed during the transcription process. The first two authors had prior experience in qualitative methods and coded the transcripts. The first author coded the first five transcripts by assigning interpretative codes to sections of text based on repeated patterns, meanings, and concepts in the transcripts to generate a draft codebook. The codebook draft was then used by the second author to code the same transcripts. The coders reviewed their independent analysis of the first five transcripts until 100% agreement was reached. During this process codes were deleted, added and clarified by the two coders to create a working codebook. The two coders each independently coded the remaining 15 transcripts. The coders met regularly to review codes, discuss text interpretations, and resolve coding discrepancies, ensuring 100% agreement on codes assigned in the final coded transcripts. During this process, the codebook acted as a dynamic document, with codes being added and modified as deemed necessary in an iterative process. Outliers were noted separately and revisited during this process. Dedoose software was used for coding, code organization, data management, and to assist in qualitative analysis, including tracking code frequency and co-occurrence.^13^ A thematic analysis was conducted to systematically examine the data and develop overarching themes.^14,15^ Individual codes were examined as well as the patterns between them to group codes into overarching themes. These themes were iteratively reviewed and woven together to create summative narratives that comprised the main findings of the study.

## 3. Results

### 3.1. Participant Characteristics

Thematic saturation was achieved at 30 participants. The results of the demographics survey are in Table 1. The average age of participants was 31.4 years old. The sample was racially and ethnically diverse with high representation from Asian, white, and Hispanic race and ethnicities. The sample was also well educated, with most completing at least a bachelor’s degree, and included greater representation from higher income ranges. All but one of the participants had health insurance.

**Table 1.** Demographic Information of Recruited Participants Who Completed Demographics Survey, 2022-2024

Demographics (N=26)	N	%
Age		
10-19 years	1	4%
20-29 years	7	27%
30-39 years	15	58%
40-49 years	3	11%
Race/Ethnicity		
Asian	8	31%
Black or African American	1	4%
Native Hawaiian/Pacific Islander	1	4%
White/Caucasian	6	23%
Hispanic/Latino	6	23%
Other	2	8%
Decline to answer	2	8%
Health Insurance status		
Insured	25	96%
Uninsured	1	4%
Education		
Less than high school diploma	1	4%
High school degree or equivalent	5	19%
Bachelor’s degree (BA, BS)	9	35%
Master’s degree (MA, MS, Med)	5	19%
Doctorate (PhD, EdD, MD)	3	11%
Trade School	2	8%
Other	1	4%
Income		
Less than 20,000	2	8%
$20,000 to $49,999	2	8%
$50,000 to $99,999	7	27%
$100,000 to $149,999	3	11%
Over $150,000	10	38%
Prefer not to say	2	8%

A diverse range of pregnancy outcomes and pregnancy intentions were represented in the study as shown in Table 2. Of the 30 participants, 11 were diagnosed with early pregnancy loss, 12 intrauterine pregnancy, and 7 ectopic pregnancy. A range of pregnancy intentions were represented (desired, undesired, unsure) with desired pregnancy being the most common (66%). Most patients had their initial ultrasound in the Emergency Department (ED) (66%, n=20) and 83% (n=25) received care in the ED for the diagnosis and/or follow-up of pregnancy of unknown location.

**Table 2.** Final Pregnancy Outcome and Pregnancy Intention of Recruited Participants, 2022-2024

​	N=30 (%)
Final Pregnancy Outcome	
EPL	11 (37%)
IUP	12 (40%)
Ectopic	7 (23%)
Pregnancy Intention	
Desired	20 (67%)
Undesired	7 (23%)
Unsure	3 (10%)
Initial Ultrasound site	
ED	20 (67%)
Clinic	7 (23%)
Planned Parenthood	3 (10%)

Through the qualitative analysis we grouped the data into four main findings: 1) anxiety and negative emotions related to not knowing the location of the pregnancy, 2) effective coping strategies including social support, individual resilience, and acceptance, 3) positive impact of empathetic and responsive providers, 4) poor access to outpatient care resulting in seeking care in the emergency department with concerns regarding long wait times and lack of privacy. The following sections will discuss these themes including pertinent quotes. Each quote is followed by information about the speaker including pregnancy intention and final outcome of the pregnancy.

### 3.2. Anxiety and Negative Emotions due to Not Knowing the Location of the Pregnancy

Participants shared feelings of substantial anxiety, confusion, and negative emotions associated with their PUL diagnosis. Many participants endorsed that they were initially confused that the location of the pregnancy could not be identified. For example, one participant said:

*“I was like, ‘What do you mean? You don't see it?’ It was a little bit of like panic, you know? Like whoa, what's happening right now?”* (Desired, early pregnancy loss)

For another participant the question of “where is the baby?” (unsure, early pregnancy loss) was a recurring phrase she returned to when describing her PUL experience.

Several participants shared feeling considerable anxiety and fear after being diagnosed with pregnancy of unknown location due to the uncertainty related to the diagnosis and sentiment of lack of control. For example, one participant said:

*“And along with that, the pain as well of not really knowing what's going on inside you. That is very, it's been very scary. And very like worrisome…it's just been very like mentally hard*.*” (Undesired, ectopic)*

In a similar vein, another participant shared her fear of having an ectopic pregnancy after being diagnosed with PUL:

“*But obviously during that week I was quite scared, you know. Knowing that there could be a bomb that could explode, that's usually the words I was using to describe that situation. I felt like there was a bomb that's implanted in my body that could explode any time.” (Desired, early pregnancy loss)*

Some patients endorsed that their anxiety surrounding their PUL diagnosis persisted even after the final outcome of the pregnancy was determined. For example, some participants endorsed feeling more anxious or cautious to attempt pregnancy again in the future. Other participants diagnosed with intrauterine pregnancies after PUL endorsed increased baseline anxiety, especially in the first trimester. For example, this participant discusses her persistent feelings of anxiety leading her to get frequent ultrasounds during the early pregnancy period:

“I freaked out until like 12 weeks…until like 14 weeks I was almost having one ultrasound per week…I was always trying to get some…medical proof… I was really scared about losing the baby… the first trimester…just gave me a lot of anxiety actually.” (Desired, IUP).

### 3.3. Effective Coping Strategies Included Social Support, Individual Resilience, and Acceptance of PUL

Participants cited having social support as beneficial, especially support from a partner, friends, or family. For example, one participant discusses how her friends helped her during this time,

“My friends, my girlfriends were very, very supportive, I would say, like three quarters of my girlfriends, all have had miscarriages before. So, being able to get that support from them from people that like have been through it before was like super duper helpful, and they would check in with me and like text me, and and call and make sure I'm okay and tell me about their experience.” (Desired, early pregnancy loss).

Many participants spoke about their experience with the PUL diagnosis describing it as a significant, impactful, and sometimes a “traumatic” life event. For example, one participant shared how she felt after being diagnosed with PUL and the follow-up period thereafter:

“I was pretty numb like, I think your mind can go to like a really dark place. I feel like I was a little traumatized by it all.” (Desired, ectopic)

Effective coping strategies included a sense of acceptance of the diagnosis of PUL and associated experiences. For many participants acceptance included expressing a sentiment of “it is what it is” related to their PUL experience. Participants who coped well also expressed associated resilience in overcoming the physical and emotional hardships related to the PUL experience. One participant, for example, expressed a sense of acceptance and resilience:

“Yeah, I mean, it was very traumatic and sad. But I've done a lot of personal healing in the past few years so I was able to, you know, just feel it and grieve it, and I'm moving through it a lot more easily than I think I would have in the past. So yeah… it is what it is.” (Desired, ectopic).

In a similar vein, another participant shared how deeply this experience impacted her at the time. She also expressed a sense of resilience and strength for overcoming this negative experience:

“Yeah like, I look back and I recovered from like extremely depressed to sadness, to right now I'm like getting back on my feet and have my own life back together. So, so yeah, it's definitely empowering.” (unsure, early pregnancy loss)

### 3.4. Empathetic and Responsive Clinicians Contributed to Positive Patient Experience With the Diagnosis of Pregnancy of Unknown Location

Regarding preferences for health clinician characteristics, patients shared positive experiences related to having an empathetic and responsive clinician. Participants appreciated clinicians who were caring with compassionate bedside manner. They also spoke well of clinicians who were prompt, responsive, and proactive in their communication, especially during the follow-up period. For example, one participant discusses how characteristics of her clicians contributed to a positive experience:

“I felt like they were all like very caring and like sincere like they really cared. They wanted to take care of me. Not just like another patient, but like to see, figure out what's going on like, ‘why is this happening. Let's do this. Let's do that. Let's try this. Do you wanna try this?’ And then so just like explore options. They're all very caring and kind of attentive and and following up with me, or referring to another doctor. So they were, I felt like everybody was really good” (desired, ectopic)

### 3.5. Poor Access to Care Resulted in Some Patients Seeking Care in the Emergency Department With Subsequent Concerns for Long Wait Times, Lack of Privacy

Many patients reported difficulty accessing outpatient care for their early pregnancy symptoms due to lack of appointment availability. Consequently, many were directed to the emergency room to receive care. One participant expressed her frustration related to difficulty accessing care through her primary care doctor (PCP) and obstetrics and gynecology (OBGYN) provider:

“I was a bit frustrated that my PCP couldn't give me any information or any advice other than go to the emergency room. And that the OBGYN office couldn't see me more urgently… all they said that, was ‘hey, we will see you at 8 weeks’” (desired, IUP).

Another participant discussed difficulty accessing care for her early pregnancy symptoms and her concern that she could not be seen outpatient a timely manner:

“I told [my PCP] like how severe the pain was at first, too, and they told me that the soonest soon I could come in, was like September 5th, or something like that. And I told them that was like way too long…And I'm glad I didn't wait…who knows what could have happened without any treatment” (undesired, ectopic).

Some participants shared negative experiences while seeking care in the emergency department for early pregnancy symptoms and PUL. Participants voiced feeling neglected, deprioritized, and overlooked. Many participants shared concern for a lack of privacy while in the emergency department. One participant, who was roomed in a hallway bed in the emergency department, shares how having a lack of privacy amplified her emotional distress. Her account illustrates a profound disconnect and contrast of her experience and emotions to the surrounding environment of the ED:

“You know I was bleeding, and I was cramping, and I was crying… But it was just surreal to be going through what I was going through in front of everybody, having no privacy… You're reminded very clearly and almost harshly, in a way that, like the world, doesn't stop for you and your problems…Like, here I am grieving my loss, but there are nurses, you know, four feet away from me, talking about what they're gonna order for lunch, or talking about like you know the date that they had, or like other patients” (Desired, early pregnancy loss).

Participants spent many hours in the emergency department while seeking care for PUL. They shared concern over long wait times and felt that this negatively impacted their experience. For example, one patient said:

“So I was in the hallway for like quite a long time, for like 3 hours…at that point I thought that I did have an ectopic pregnancy, and I was like, well, this could go wrong at any point… [I] wish I had like had more like urgent medical staff see me, cause it feel like it took a long time to get seen. And then for labs, and it was just an entire day's process (unsure, IUP).”

Another patient discusses her frustration due to lack of closure from the PUL diagnosis, which was compounded by her concern for a long wait time in the ED:

“The only thing that like bothered me and concerned me was the length of time that I was in there…it was a long day to go home still not knowing anything and feeling lost” (desired, early pregnancy loss).”

Other participants shared their frustrations with the longer wait times in the emergency department as they felt the medical care they received could have been completed outpatient. She said:

“I wish there was a way to get that ultrasound without being in the ER, if there was a way to make that happen like that would have been better. Cause I think the only thing they did in the ER was the ultrasound and the blood tests which could have been done like outpatient so that would have saved me 8 hours in the ER…they would have told me the same thing, you know it's inconclusive, and just keep drawing your labs” (desired, early pregnancy loss)

## 4. Discussion

The findings of our study suggest that patients may experience anxiety, confusion, and negative emotions related to the PUL diagnosis stemming from the uncertainty, lack of control and lack of closure inherent in the diagnosis. Similar to these findings, other studies have documented anxiety and negative emotions related to other early pregnancy diagnoses including pregnancy of uncertain viability, first trimester vaginal bleeding, ectopic pregnancy, and early pregnancy loss.^4,5,16,17^ The results of our study support that these negative emotions may persist even after the pregnancy of unknown location episode resolves and can affect patients’ experiences of an ongoing intrauterine pregnancy or impact thoughts on future pregnancies. Additionally, our study illustrated a larger sentiment that the diagnosis and follow-up of PUL can be a major life event described as “traumatic” by participants and require time and emotional labor to overcome. Similarly, a systematic review on patients who had a miscarriage or recurrent miscarriages found that this experience is a significant life event for patients and their partners.^18^

This study also documented that some participants had negative experiences while seeking care for early pregnancy symptoms in the emergency department. Other studies have also documented patient dissatisfaction with care for miscarriage in the ED due to lack of emotional support.^5^ In another study, patients presenting with miscarriage to the ED were less satisfied with their care and reported a lack of clarity surrounding their diagnosis, inefficient care, and a mixed experience with health care provider sensitivity.^19^

The strengths of this study include its racially diverse sample with large representation from Hispanic, Asian, and white participants. The study sample also contained a variety of different pregnancy intentions, which have not been represented in prior study populations. Our study also included a diverse range of PUL final outcomes, including ectopic pregnancy which is a rare pregnancy outcome. Inclusion of participants across these groups was important, as the primary study findings were consistent across these varied clinical and experiential contexts. This consistency strengthens the credibility and potential transferability of our findings. Lastly, we recruited participants who received care from outpatient clinics, emergency departments, and at a Planned Parenthood, therefore making the results more transferable to various clinical settings.

Limitations of the study include decreased representation of lower socioeconomic status and Black participants. This study included a higher proportion of participants who were more educated, wealthy, and had health insurance. It is telling that despite having these resources and attributes usually associated with better access to healthcare, our study sample still reported barriers to accessing care when experiencing PUL. Among populations of patients without socioeconomic advantages, accessing care for PUL may be even more challenging. Additionally, this study mostly recruited from one region of the United States. Consequently, some of the findings may not be transferable to other regions and institutions which may have different infrastructure and processes for caring for patients with early pregnancy symptoms. Future studies could include participants from demographic groups not as well represented in the current sample and from additional regions across the United States.

The findings of this study provide many insights to improve the care of patients diagnosed with pregnancy of unknown location through changes in provider behavior, new interventions, and institutional change. First, clinicians should be aware of patients’ experiences with PUL and strive to be more empathetic, proactive, and responsive when caring for these patients. Future interventions could be created to better train clinicians on how to offer emotional support to patients with PULs.

The study findings also suggest that patients could benefit from additional mental health support both while diagnosed with PUL and after the episode has resolved. Future interventions could incorporate the findings of this study to offer a personalized approach to provide better psychosocial support for patients with this diagnosis.

The fourth theme of this study, difficulty accessing care and negative patient experiences in the emergency department, suggests that institutions and clinicians could consider creating more patient-centered policies and infrastructure to improve the care of patients with pregnancy of unknown location. In this vein, institutions could consider adopting an alternative algorithm to approach management of PUL. For example, Flynn et al. developed a patient-centered protocol for the management of PUL that prioritizes and personalizes recommendations based on pregnancy desiredness.^20^

Regarding improvements in infrastructure, it is important to consider early pregnancy assessment units (EPAUS). EPAUs are specialized clinics, most common in the United Kingdom and Canada, that diagnose and care for patients with early pregnancy complications.^21^ Higher use of these units has been associated with increased access to care, improved quality of care, overall cost-savings, reduced emergency department utilization, and improved patient-centered care. A similar model to early pregnancy assessment units has been adopted in the United States, providing comprehensive and streamlined services for early pregnancy patients.^21^ The findings of this study suggest that more widespread adoption of early pregnancy units in the United States could fill a needed gap in healthcare by improving access and quality of care for patients with PULs.

## 5. Conclusion

This study provides insight into patients’ experiences with PUL, specifically anxiety and negative emotions related to diagnostic uncertainty in early pregnancy. It also highlights the importance of having supportive clinicians and the optimal setting for PUL care. The findings of this study provide valuable insights which could inform interventions to improve patients’ experiences with PUL at both the clinician and institutional level. We hope that this study may encourage institutions to examine their particular processes for caring for these patients and find potential opportunities within their own infrastructure to address the common concerns raised by patients in this study.

## Appendix

### Appendix 1 PEPUL Study Interview Guide

1. Tell me about experience receiving the ultrasound results indicating a pregnancy of unknown location? *(Prompts: Thoughts/feelings when hearing ultrasound findings. Experience of processing this news)*
2. During follow-up: Tell me about your pregnancy of unknown location follow-up?
(Prompts: experiences/feelings/emotions about the follow up process (HCG, D&C procedures, fear of having a tubal pregnancy))
3. Resolution –Tell me about the final outcome after follow-up?
(Prompts: experiences/feeling/emotions regarding this final outcome-ectopic pregnancy, intrauterine pregnancy, early pregnancy loss)
4. Support? – Who provided you emotional supported you during the time you had pregnancy of unknown location? *(Prompts: partner, friend, case manager)*
5. Coping –Tell me about how you coped during the time you had a pregnancy of unknown location?
6. Knowledge? – Did you have any knowledge of this diagnosis prior to your experience? how did you learn about it? *(family, friends, medical team, websites)*
7. Impact: Tell me about your how experience with pregnancy of unknown location has impacted your pregnancy intention or thoughts about your fertility?
8. Feedback: Tell me about how medical staff impacted your experience?
(Prompt: What aspects of your care were positive? What aspects were negative?)
9. Feedback: Do you have any suggestions or feedback for the medical staff when caring for patients with pregnancy of unknown location?
10. Do you have any suggestions/feedback for logistical or system change to improve this experience for future patients with pregnancy of unknown location?
